# Analysis of L Antigen Family Member 3 as a Potential Biomarker and Therapeutic Target Associated With the Progression of Hepatocellular Carcinoma

**DOI:** 10.3389/fonc.2022.813275

**Published:** 2022-03-31

**Authors:** Qianhui Chen, Xinyu Lu, Jiayi Xie, Na Ma, Weikang Xu, Zhiming Zhang, Xuan Huang, Hongyan Liu, Jinlin Hou, Xiaoyong Zhang, Wei Zhu

**Affiliations:** ^1^ State Key Laboratory of Organ Failure Research, Guangdong Provincial Key Laboratory of Viral Hepatitis Research, Department of Infectious Diseases, Nanfang Hospital, Southern Medical University, Guangzhou, China; ^2^ Department of Pathology, The First People’s Hospital of Foshan, Foshan, China

**Keywords:** hepatocellular carcinoma, L antigen family member 3, biological function, therapeutic target, biomarker, tumorigenicity

## Abstract

**Background:**

Hepatocellular carcinoma (HCC) is the third cause of cancer-related deaths worldwide. L antigen family member 3 (LAGE3) is a prognostic biomarker and associated with progression in a variety of tumors. However, little has been reported about the role and potential mechanism of LAGE3 in HCC.

**Methods:**

The clinical value and function of LAGE3 in HCC were obtained from multiple online databases. The potential functions and pathways of LAGE3 in HCC were analysed by R package of “clusterProfiler”. LAGE3 knockdown cells were constructed in HepG2, HuH7 and MHCC97H cell lines, respectively. The biological roles of LAGE3 were examined by *in vitro* and *in vivo* experiments.

**Results:**

LAGE3 was upregulated in HCC tissues compared with normal tissues, and high expression of LAGE3 was significantly associated with several clinical characteristics and indicated a worse prognosis of HCC. The co-expressed genes of LAGE3 could be enriched in the mTOR signaling pathway in HCC. LAGE3 was upregulated in HCC cell lines. Functionally, knocking down LAGE3 expression not only increased apoptosis and inhibited growth rate, cell death mediated by T cells, colony formation, migration and invasion ability of HCC cell lines *in vitro*, but also reduced the progression of HCC in the subcutaneous xenotransplanted tumor model.

**Conclusion:**

Our results suggested that LAGE3 served as an oncogenic factor of HCC and could be a potential biomarker and therapeutic target for HCC.

## Introduction

Hepatocellular carcinoma (HCC), the main pathological type of primary liver cancer worldwide, is the sixth most common cancer and the third leading cause of cancer-related mortality (1, 2). Currently, multimodal therapies, including surgical, locoregional, and systemic therapies, have made great progress in recent decades; however, the efficacy of any treatment approach depends on many parameters, such as the degree of underlying liver disease, the size and extent of the tumor, the presence of vascular invasion, and the patient’s performance status (2). Thus, the outcomes of curing HCC and the five-year relative overall survival (OS) rate remain unsatisfactory.

In recent years, based on the new concept of “precision medicine”, advancements in molecular pathology and targeted therapies have significantly improved the OS rate of HCC patients. In general, key genes that drive carcinogenic effects can be used as biomarkers and therapeutic targets. The tyrosine kinases vascular endothelial growth factor receptor (VEGFR), platelet-derived growth factor receptor (PDGFR), and fibroblast growth factor receptor (FGFR) are molecules commonly reported for pathological detection and therapeutic targets of HCC (3–5). Although targeted drugs have prolonged median survival of patients (3–5), molecularly targeted therapies in HCC remain challenging, particularly for the low response ratio and occurrence of drug resistance. Therefore, it is necessary to develop novel biomarkers as potential targets for diagnosis, prognosis prediction, and treatment for HCC patients.

L antigen family member 3 (LAGE3), a 14,804-Da intracellular protein, is known as a component of the kinase, endopeptidase and other proteins of small size/endopeptidase-like and kinase associated to transcribed chromatin (KEOPS/EKC) complex (6, 7). LAGE3 protein plays an important role in the regulation of RNA polymerase II-mediated positive transcription, translation as well as tRNA threonyl carbamoyl adenosine metabolic processes (8, 9). Previous studies have found that LAGE3 is one of the top-ranked up-regulated RNA modification-related proteins in multiple human cancers (10). In addition, studies have found that LAGE3 is a prognostic biomarker associated with levels of immune infiltration in the microenvironment of clear cell renal cell cancer, colorectal cancer, malignant pleural mesothelioma, breast cancer (BC), skin cutaneous melanoma, and papillary thyroid cancer (PTC) (11–16). Furthermore, studies have shown that knocking down LAGE3 expression may significantly reduce the proliferation, migration, and invasion capacity of PTC and BC cell lines (14, 16). Similarly, Xing et al. also found that LAGE3 could promote cell proliferation, migration, and invasion of HCC by facilitating the JNK and ERK signaling pathway (17). However, previous study only used one or two HCC cell lines to verify the role and partial regulatory mechanisms of LAGE3. Thus, the biological function of LAGE3 protein in HCC requires further validation. More studies are needed for its clinical value and potential mechanisms as well.

In this study, firstly, based on multiple databases, we comprehensively assessed the expression and prognostic significance of LAGE3 gene in HCC. We also identified a prominent correlation between LAGE3 and immune cells in HCC. Besides, we found that the co-expressed genes of LAGE3 could be enriched in the mTOR signaling pathway. To further investigate the biological function of LAGE3 in HCC, we explored the effects of knocking down LAGE3 expression on cell proliferation, apoptosis, cell cycle, migration, and invasive malignant behaviors in HepG2, HuH7 and MHCC97H cell lines *in vitro.* Moreover, the function of LAGE3 on HCC progression was further identified by subcutaneous xenotransplanted tumor models of HepG2, HuH7 and MHCC97H cell lines in nude mice. In addition, LAGE3 knockdown could inhibit the HCC cell death mediated by cytotoxic T cells. Our results suggested that LAGE3 served as an oncogenic factor of HCC, and could be a potential biomarker and therapeutic target.

## Materials And Methods

### Patient Data Sets

mRNA expression data (374 samples, Workflow Type: HTSeq-FPKM) and clinical information from The Cancer Genome Atlas (TCGA) database (https://cancergenome.nih.gov) were analyzed by the online tool (https://www.xiantao.love/products). Samples were excluded with (1) “0” gene expression value and (2) insufficient survival information. A total of 373 patients with HCC having complete data relative to the corresponding clinical features were enrolled in this study. The data was used to compare the levels of LAGE3 mRNA expression in normal and HCC tissue. The correlation between LAGE3 expression and clinical characteristics or immune cell infiltration of HCC patients was also investigated using these data. In addition, receiver operating characteristic (ROC) curve analysis of LAGE3 gene expression was used to evaluate the diagnostic value of this gene. Finally, Kaplan-Meier curves with the log-rank test were performed to evaluate patient survival.

### Oncomine Database and Tumor Immune Estimation Resource (TIMER) Database Analysis

The Oncomine database has compiled 86,733 cancer tissues and normal tissues and 715 gene expression data sets into a single comprehensive database designed to facilitate data mining efforts (https://www.oncomine.org/resource/login.html) (18). The TIMER database includes 10,897 samples across 32 cancer types from The Cancer Genome Atlas (TCGA) to estimate the abundance of immune infiltrates (https://cistrome.shinyapps.io/timer/) (19). Therefore, we used both databases to investigate LAGE3 expression in different types of cancer. Moreover, the correlations between the expression of LAGE3 and specific immune infiltrating cell subset markers were also analyzed using the TIMER database.

### Protein Expression Analysis

The immunohistochemistry (IHC) images of normal and HCC tissue were obtained from the Human Protein Atlas (HPA) (20) and were used to identify subcellular localization and to assess LAGE3 protein expression.

### Protein-Protein Interaction (PPI) Analysis

The Retrieval of Interacting Genes/Proteins (STRING) website (https://version-11-0b.string-db.org/) was used to construct the PPI information of LAGE3. A confidence score > 0.7 was considered significant.

### Functional Enrichment Analysis

The HTseq-FPKM data of liver hepatocellular carcinoma (LIHC) was obtained from UCSC XENA database (https://xenabrowser.net/datapages/), including 424 samples. The correlation analysis between LAGE3 and other genes was performed by the function “cor.test” implemented in R with the Spearman method. Firstly, genes were filtered by *p* < 0.05 and *r* > 0.5. Then the Gene Ontology (GO) analysis were conducted by “clusterProfiler” package. The Gene Set Enrichment Analysis (GSEA) of Kyoto Encyclopedia of Genes and Genomes (KEGG) was analysed by *q* < 0.1 using “clusterProfiler” package (21). The plots were visualized using “ggplot2” package.

### Gene Expression Profiling Interactive Analysis (GEPIA) Database Analysis

The GEPIA (http://gepia.cancer-pku.cn/index.html) is an online database which consists of RNA-seq data from 9736 tumors and 8587 normal samples in TCGA and Genotype-Tissue Expression (GTEx) data. The GEPIA database was used to further verify the markers associated with immune cell infiltration, which were significantly correlated with LAGE3 expression in the TIMER database.

### Cell Culture

The human normal hepatic cell line L02 and human HCC cell lines HepG2, HuH-7, and MHCC97H were obtained from the Shanghai Cell Bank of the Academy of Chinese Sciences and Liver Cancer Institute, Zhongshan Hospital, Fudan University (China). All cell lines were cultured in complete Dulbecco’s Modified Eagle’s Medium (DMEM) (Gibco, USA) supplemented with 10% fetal bovine serum (Gibco), 100 U/mL penicillin (Gibco) and 0.1 mg/mL streptomycin (Gibco). All cells were cultured in a 37°C, 5% CO_2_ humidified atmosphere incubator.

### Small Interfering RNA (siRNA) Transfection

Three siRNAs targeting LAGE3, including si-LAGE3-1: GAAUGCGGCCGCACAUAUUTT (forward) and AAUAUGUGCGGCCGCAUUCTT (reverse); si-LAGE3-2: CCUGGUCGUCCGCUGGAAATT (forward) and UUUCCAGCGGACGACCAGGTT (reverse); and si-LAGE3-3: GCGGACCAUGCAGCGCUUUTT (forward) and AAAGCGCUGCAUGGUCCGCTT (reverse), as well as a scrambled siRNA for the negative control (NC), were synthetized by GenePharma (China). When the cells reached the logarithmic growth phase, approximately 1×10^5^ cells/well were seeded into the 6-well plates for 24 hours before transfection. Based on the manufacturer’s instructions, Lipofectamine RNAiMAX (Invitrogen, USA) was then mixed with siRNA for the transfection of cell lines. The cells were harvested 48–72 hours after transfection and examined the transfection efficiency of siRNAs by quantitative Real-time polymerase chain reaction (qRT-PCR).

### Short Hairpin RNAs (shRNAs) Lentivirus Infection

Lentivirus-shRNA of LAGE3 (lenti-shLAGE3) and negative control (lenti-NC) were designed and constructed by GenePharma. The HCC cell lines were infected by lenti-shLAGE3 or lenti-NC according to the manufacturer’s instructions. The oligonucleotide sequences of shLAGE3 and negative control were: 5’-GACTGTCGCCTGCTCCGAATT-3’ and 5’-TTCTCCGAACGTGTCACGT-3’ respectively.

### qRT-PCR of Cell Lines

Total RNA was isolated using the EZ-press RNA Purification Kit (EZBioscience, USA) following the manufacturer’s instructions. The cycles of threshold (Ct) were detected by qRT-PCR using the LC480 system and the EZ-press One Step qRT-PCR Kit (EZBioscience). The relative expression of LAGE3 was normalized according to expression of β-actin. The gene expression of the gene of interest was presented as fold change, which was analyzed using the 2^-ΔΔCt^ method. The primer sequences used were as follows: for LAGE3, forward, 5’-GTTGATGACGGAAATTCGGAGCAG-3’ and reverse, 5’-ACCAAAGGGTGGTTGGGAAGGAT-3’; and for β-actin, forward, 5’-TCCCTGGAGAAGAGCTACGA-3’ and reverse, 5’-AGCACTGTGTTGGCGTACAG-3’.

### Cell Counting Kit-8 (CCK8) Assay

To determine the proliferative activity of liver cancer cells, the CCK-8 (Takara, Japan) was used in this study. Forty-eight hours after transfection, the proliferation ability of HepG2, MHCC97H, or HuH-7 cells was detected. Briefly, 100 μL of cell suspension was seeded into a 96-well plate (1000 cells/well) in triplicate and incubated at 37°C for 24, 48, 72, or 96 hours respectively. Then 10 μL CCK-8 reagent was transferred to each well. After 1.5 hours, the OD value was detected at 450 nm using Gen5 software (Biotek, USA). The experiment was repeated three times independently and showed the same results.

### Colony Formation Assay

For the colony formation assay, after 48 hours of cell transfection, 2.5 mL cell suspension was transferred to each well in a 6-well plate (about 1000 cells/well), which was then incubated at 37°C, 5% CO_2_ for 10–14 days until colonies emerged. Next, the supernatant was removed and cells were gently washed twice with PBS. After fixing with 4% paraformaldehyde for 30 min, the cells were stained with 0.1% crystal violet for 20 min. The plate was carefully washed with running water and then dried naturally. The size and number of clones were determined by Image J software.

### Cell Apoptosis and Cell Cycle Assay

For detecting cell apoptosis, transfected cells were collected, washed once with PBS, and centrifuged at 1000 rpm for 5 min. Then cells were suspended in 200 µL binding buffer with 5 µL Annexin-V-FITC and 5 µL 7-AAD (BioLegend, USA) for 15 min at room temperature in the dark. The FACS CantoTM II Flow Cytometer (BD Biosciences, USA) was used to analyze the proportion of apoptotic cells within 1 hour.

To assess cell cycle, the transfected cells were washed twice with PBS and fixed with 70% precooled ethanol at 4°C overnight. The cells were then washed twice again with PBS and cultured with RNase buffer and propidium iodide (PI) away from light for 30 minutes at 4°C. The changes in G0/G1, G2/M, and S phases of the cell cycle was detected by flow cytometry.

### Cell Migration and Cell Invasion Assay

To evaluate the capacity of cell migration, 100 μL transfected cell suspension containing 5×10^4^ cells was seeded in the upper chamber of an 8 μm microporous filter (Corning, USA). The lower chamber of the Transwell was filled with 700 μL complete medium. The plate was incubated for 48 hours at 37°C, 5% CO_2_. After removal of the non-migrated cells in the upper chamber, the cells on the polycarbonate microporous membrane at the bottom of the chamber were fixed with polyformaldehyde for 30 min. Next, the cells were stained with 0.1% crystal violet for 15–20 min. The invasion assay followed a similar protocol as the migration assay as described above, except that the Transwell chambers were loaded with 40 μL Matrigel (BD Biosciences) at 37°C for 4 hours before seeding cells. The number of migrated or invaded cells were assessed by microscope (IX73, Olympus, Japan) and ImageJ software.

### Wound Healing Assay

To further study the cell migration, transfected cells were seeded in the 6-well plate. When the cells reached 90% confluence, the cell monolayers were wounded using a pipette tip and then washed with PBS three times. The cells were cultured for another 48 hours after adding serum-free medium. The cell migration was assessed by measuring the scratch area using Image J software.

### Western Blotting Analysis

For immunoblotting, protein samples from cells in each group were collected with Blue Loading Buffer Pack (7722S, CST, USA). After heating at 100°C for 10 minutes, the protein lysates were separated by sodium dodecyl sulfate polyacrylamide gel electrophoresis (SDS-PAGE) and transferred to the PVDF membrane (Millipore, USA) to be incubated with the following primary antibodies at 4°C overnight: anti-Bax antibody (1:1000; bs-0127M; Bioss, China), anti-Bcl-2 antibody (1:1000; ab32124, Abcam, USA), anti-caspase 3 antibody (1:1000; 9662S; CST), anti-cleaved-caspase 3 antibody (1:1000; 9664S; CST), anti-β-Catenin antibody (1:1000; 8480S; CST), anti-E-Cadherin antibody (1:1000; 3195S; CST), anti-N-Cadherin antibody (1:1000; 22018-1-AP; ProteinTech, USA), and anti-β-actin antibody (1:1000; 4970L; CST). Next, the membranes were incubated with anti-rabbit or anti-mouse immunoglobulin G conjugated with horseradish peroxidase for 1 h at room temperature. Finally, the ECL detection kit (Beyotime Biotechnology, China) was used to detect the protein bands. The bands were photographed and quantified using the UVP BioSpectrum AC image system (Upland, USA) and ImageJ software respectively.

### Construction of Xenograft Models

The animal study was reviewed and approved by the Animal Ethics Committee of Nanfang Hospital of Southern Medical University (China). Thirty-six male BALB/c-nu nude mice (aged 3–5 weeks, weighing 16–20 g) were acquired from the Hunan SJA Laboratory Animal Co., Ltd (China). The HepG2, Huh7 and MHCC97H cells were infected with lentiviruses expressing empty vector or LAGE3-shRNA respectively. Subsequently, the infected cells were subjected to puro (3–6 µg/mL) screening and a 0.05 mL volume of cell suspension (5×10^6^ cells for each mouse) in the logarithmic growth phase was subcutaneously injected into the nude mice (n  =  6 for each group) at the right hind limb groin. The growth of xenografts was monitored, by taking tumor length (L) and width (W) measurements to calculate the tumor volume (V). The following formula was used: V (mm3) = 0.5 × L × W^2^. The observation time ended when the maximum tumor volume reached about 1000 mm3. The corresponding growth curve of tumor was drawn. At the end of the experiment, the mice were euthanized and the excised tumors were weighed and photographed.

### IHC

Tumor tissues were fixed with 4% paraformaldehyde for 24 hours at room temperature, embedded in paraffin and sectioned into 4μm-thickness. Following dewaxing and rehydration, heat induced antigen retrieval was performed using Tris/EDTA buffer pH 8.0, microwaved for 8-15 minutes. Then the endogenous peroxidase activity was blocked with 3% hydrogen peroxide for 15 minutes. Subsequently, the slides were incubated overnight with antibodies specific for Ki67 (1:4000; 27309-1-AP; ProteinTech) at 4˚C after blocking the non-specific binding sites with the goat serum for 90 minutes. Then, anti-rabbit/mouse IgG-HRP-linked secondary antibody (GK500710, genetech, USA) was applied for 1 hour at room temperature. And the sections were developed with 3-diaminobenzidinetetrahydrochloride for 5-10 minutes. The Mayer’s hematoxylin was applied for 5 minutes to stain nuclei. Photographs of representative fields were captured under BX63 microscope (Olympus) and analysed by ImageJ software.

### Cancer Cell Death Mediated by T Cells

The preparation method of AFP TCR-T cells was according to our previous study (22). The ratio of AFP specific T cells was detected by flow cytometry with anti-mouse TCR vβ-FITC and anti-human CD8-PerCP (BioLegend). Then the Mock or AFP TCR-T cells were co-cultured with HepG2 cells, which were infected with lentiviruses expressing empty vector or LAGE3-shRNA at an effector-to-target cell (E:T) ratio of 2:1. Three hours later, the immunofluorescence stain of HepG2 cells was done with anti-rabbit cleaved-caspase3 (9664S, CST) at 1:400 and AlexaFluor 488-conjugate goat anti-rabbit IgG (ZF0511, ZSGB-BIO, China) at 1:1000. After 12 h of coculture, the cytotoxicity of T cells was detected by measuring the lactate dehydrogenase (LDH) activity according to the manufacturer’s protocol (Promega, USA). And the IFN-γ and TNF-α levels in the culture supernatant was detected using enzyme-linked immunosorbent assay (ELISA) kit (Biolegend).

### Statistical Analysis

The Wilcoxon signed-rank test and logistic regression were performed to analyze the association between LAGE3 and clinical features or immune cell infiltration in HCC. Kaplan-Meier analysis was used to compare the OS rate between the high and low LAGE3 gene expression groups using the p-value determined in the log-rank test. ROC curve was used to determine the diagnostic value of LAGE3 gene expression, with the area under the ROC curve applied as the diagnostic value. All statistical analyses were performed using R statistical software (version 3.6.3 and 4.1.2). The data relative to the *in vitro* and *in vivo* experiments were analyzed by GraphPad 9.0. The Mann–Whitney U test or student’s t-test were used to compare differences between the LAGE3 knockdown groups and NC groups. A *p*-value < 0.05 was considered statistically significant.

## Results

### Assessment of LAGE3 Expression in Different Cancers and Normal Tissues

**Figure 1:**
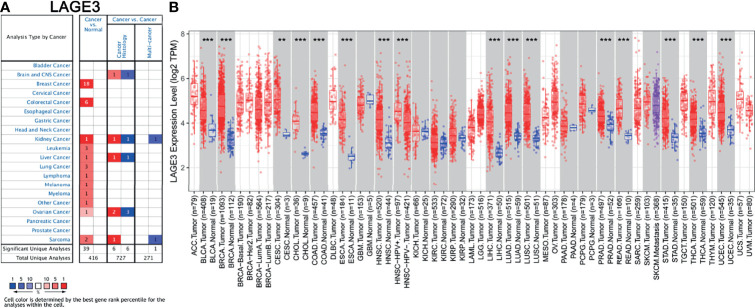
Assessment of LAGE3 expression in different cancer and normal tissues. **(A)** Expression of LAGE3 in different types of human cancers in the Oncomine database. **(B)** Expression of LAGE3 in different types of human cancers in the TIMER database. **P < 0.01, ***P < 0.001.

We first assessed the expression of *LAGE3* in multiple tumor and normal tissue types using the Oncomine database. The results revealed that the expression of *LAGE3* was elevated, compared with normal controls in breast cancer, colorectal cancer, kidney cancer, leukemia, liver cancer, lung cancer, lymphoma, melanoma, myeloma, and sarcoma respectively (**Figure 1A**). We further used the TIMER database to verify how *LAGE3* expression differed in specific tumor types. The expression of *LAGE3* was significantly elevated, compared with normal controls in bladder urothelial carcinoma (BLCA), breast invasive carcinoma (BRCA), cervical squamous cell carcinoma (CESC), cholangiocarcinoma (CHOL), colon adenocarcinoma (COAD), esophageal carcinoma (ESCA), head and Neck squamous cell carcinoma (HNSC), LIHC, lung adenocarcinoma (LUAD), lung squamous cell carcinoma (LUSC), prostate adenocarcinoma (PRAD), rectum adenocarcinoma (READ), stomach adenocarcinoma (STAD), thyroid carcinoma (THCA), and uterine corpus endometrial carcinoma (UCEC) (**Figure 1B**).

### Baseline Characteristics of Samples

We next used mRNA expression data and clinical information from TCGA database to determine whether LAGE3 expression was relevant to the clinical features, diagnostic value and prognosis in HCC patients. The clinical characteristics of patients including age, sex, race, TNM stage, pathologic stage, and vascular invasion were collected and summarized in **Table 1**. A total of 121 female patients and 253 male patients were analyzed in this study, including 185 patients of white race and 177 non-white patients. In terms of HCC pathological stage, 173 patients were classified as stage I (49.4%), 87 patients were stage II (24.9%), 85 patients were stage III (24.3%), and 5 patients were stage IV (1.4%). The tumor status included 202 tumor-free patients (56.9%) and 153 patients with tumor (43.1%). The T stage distribution included 49.3% T1 (n = 183), 25.6% T2 (n = 95), 21.6% T3 (n = 80), and 3.5% T4 (n = 13) patients. A total of 110 patients (34.6%) presented the status of vascular invasion.

**Table 1:** Clinical characteristics of the HCC patients.

Clinical characteristics	Levels	N	%
Age (years)	<=60	177	47.5
	>60	196	52.5
Gender	Female	121	32.4
	Male	253	67.6
Race	Asian	160	44.2
	Black or African American	17	4.7
	White	185	51.1
T stage	T1	183	49.3
	T2	95	25.6
	T3	80	21.6
	T4	13	3.5
N stage	N0	254	98.4
	N1	4	1.6
M stage	M0	268	98.5
	M1	4	1.5
Pathologic stage	Stage I	173	49.4
	Stage II	87	24.9
	Stage III	85	24.3
	Stage IV	5	1.4
Tumor status	Tumor free	202	56.9
	With tumor	153	43.1
Residual tumor	R0	327	94.8
	R1	17	4.9
	R2	1	0.3
Histologic grade	G1	55	14.9
	G2	178	48.2
	G3	124	33.6
	G4	12	3.3
AFP (ng/ml)	<=400	215	76.8
	>400	65	23.2
Albumin (g/dl)	<3.5	69	23
	>=3.5	231	77
Child-Pugh grade	A	219	90.9
	B	21	8.7
	C	1	0.4
Prothrombin time	<=4	208	70
	>4	89	30
Fibrosis ishak score	0	75	34.9
	1/2	31	14.4
	3/4	28	13
	5/6	81	37.7
Vascular invasion	No	208	65.4
	Yes	110	34.6

### High LAGE3 Expression in HCC Tissue

**Figure 2:**
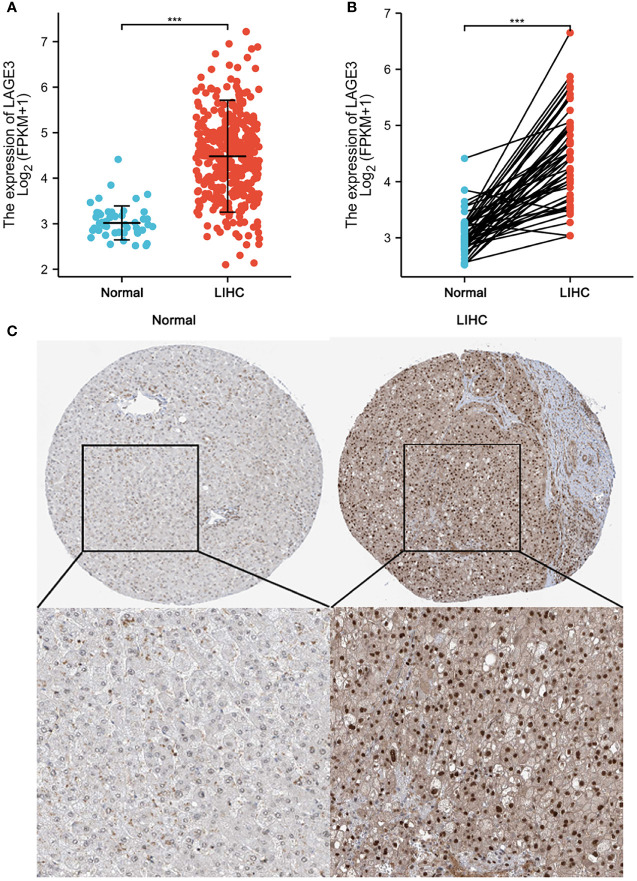
High LAGE3 expression in HCC tissue. **(A)** The level of LAGE3 mRNA expression in normal and LIHC tissues. **(B)** The level of LAGE3 mRNA expression in paired tissues. **(C)** Representative immunohistochemistry images and detailed information of LAGE3 expression in normal liver tissues and HCC tissues from the HPA. LIHC, liver hepatocellular carcinoma. ***P < 0.001.

To assess the level of LAGE3 expression in HCC patients, we compared the mRNA expression level in HCC samples to that in normal liver tissues according to TCGA database. LAGE3 mRNA expression was significantly higher in HCC tissues than in normal tissues (**Figure 2A**). The results were verified in HCC tissues and in paired normal liver tissues (**Figure 2B**). In addition, the results also showed that LAGE3 protein expression was significantly up-regulated in HCC tissues compared with normal liver tissues by analyzing IHC images obtained from the HPA (**Figure 2C**).

### Correlation of LAGE3 Expression With Clinical Features, Diagnostic Value and Prognosis in HCC Patients

**Figure 3:**
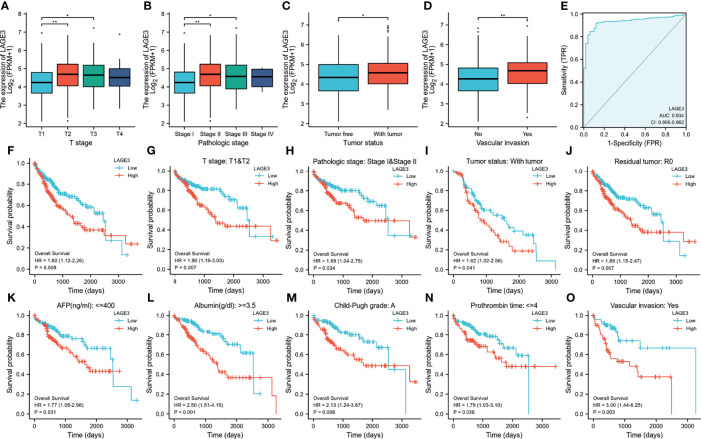
Correlation between LAGE3 expression and clinical features, diagnostic value and prognosis of LAGE3 expression in HCC patients. **(A-D)** In the TCGA database, LAGE3 expression in the different status of **(A)** T stage; **(B)** pathologic stage; **(C)** tumor status, and **(D)** vascular invasion. The data were analyzed by Wilcoxon signed-rank test. *P < 0.05, **P < 0.01. **(E)** ROC curve for LAGE3 in normal liver tissue and HCC. Kaplan-Meier curves showing the overall survival linked to LAGE3 expression in HCC. **(F)** Based on TCGA datasets, Kaplan-Meier curve for LAGE3 in all HCC patients; **(G–O)** In the TCGA database, subgroup analysis for **(G)** T1/T2; **(H)** pathologic stageI/II; **(I)** with tumor of tumor status; **(J)** R0 of residual tumor; **(K)** AFP no more than 400 ng/mL; **(L)** albumin more than 3.5 g/dL; **(M)** Child-Pugh grade A; **(N)** prothrombin time no more than 4 seconds and **(O)** with vascular invasion.

The correlation between LAGE3 expression and clinical features in patients with HCC is shown in **Figure 3**. High expression of LAGE3 was significantly correlated with T stages, pathologic stages, tumor status, and vascular invasion (**Figures 3A** **–D**). In addition, we conducted ROC curve analysis using LAGE3 gene expression data to evaluate the diagnostic value of this gene. As shown in **Figure 3E**, the area under the curve (AUC) was 0.934, which indicated LAGE3 had high diagnostic value in HCC.

We also used Kaplan-Meier survival analysis to assess how LAGE3 expression related to prognosis in HCC. We found that high LAGE3 expression was associated with poor prognosis (**Figure 3F**). We also performed subgroup analysis by different clinical features as shown in **Figures 3G** **–O**. High LAGE3 expression was significantly associated with poor prognosis in HCC cases and was correlated with different clinical features including T stage I/II, pathologic stage I/II, with tumor of tumor status, R0 of residual tumor, AFP no more than 400 ng/mL, albumin more than 3.5 g/dl, Child-Pugh grade A, prothrombin time no more than 4 seconds, and with vascular invasion.

### Constructing PPI Network of LAGE3

**Figure 4:**
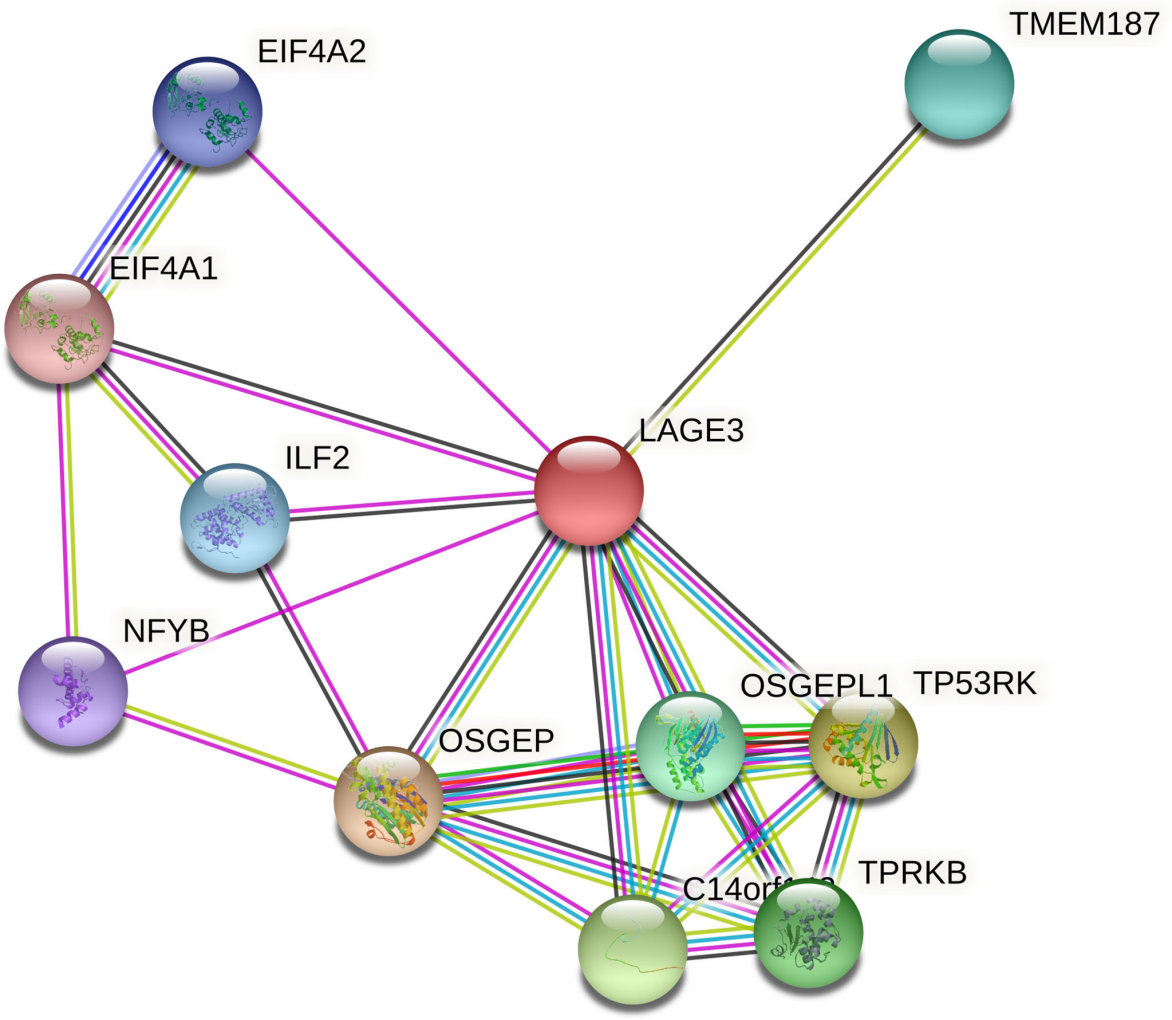
PPI network of LAGE3 protein by the STRING online tool. Top 10 LAGE3-interaction proteins in HCC tissue.

Functional interactions between proteins are necessary to better understand the complex molecular mechanisms active in malignant tumors. Therefore, we used STRING to analyze the PPI network of LAGE3 protein to determine their interactions in the progression of HCC. The top 10 proteins and corresponding gene names, annotations, and scores are listed in **Figure 4** and **Table 2**. These genes included: OSGEP, TP53RK, C14orf142, TPRKB, OSGEPL1, TMEM187, ILF2, EIF4A2, NFYB, and EIF4A1 (**Figure 4** and **Table 2**).

**Table 2:** Predicted functional partners of LAGE3 protein.

Gene symbol	Annotation	Score
OSGEP	Probable tRNA N6-adenosine threonylcarbamoyltransferase;	0.999
TP53RK	TP53-regulating kinase;	0.995
C14orf142	EKC/KEOPS complex subunit GON7;	0.991
TPRKB	EKC/KEOPS complex subunit TPRKB;	0.987
OSGEPL1	Probable tRNA N6-adenosine threonylcarbamoyltransferase, mitochondrial;	0.912
TMEM187	Transmembrane protein 187;	0.908
ILF2	Interleukin enhancer-binding factor 2;	0.857
EIF4A2	Eukaryotic initiation factor 4A-II;	0.847
NFYB	Nuclear transcription factor Y subunit beta;	0.835
EIF4A1	Eukaryotic initiation factor 4A-I;	0.830

### The Potential Functions and Pathways of LAGE3 in HCC

**Figure 5:**
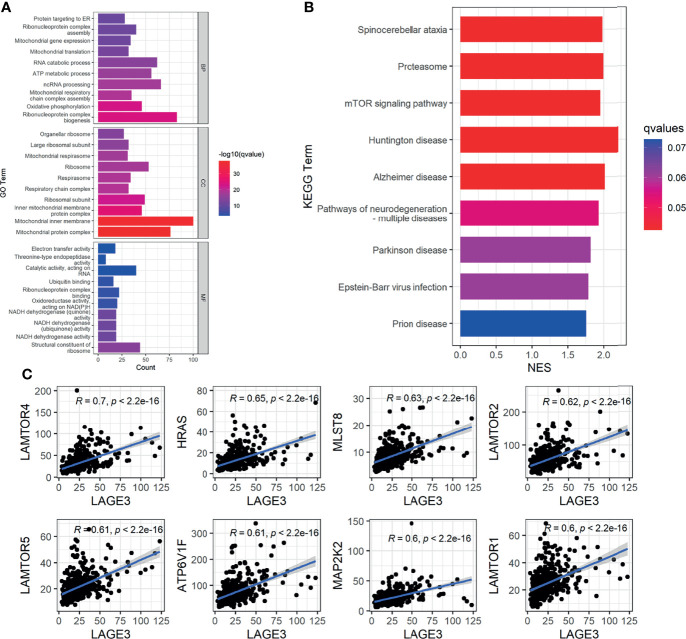
The potential functions and pathways of LAGE3 in HCC. **(A)** Top 10 terms of each subtype of GO enrichment analysis by genes co-expressed with LAGE3 in HCC. **(B)** GSEA-KEGG enrichment analysis by genes co-expressed with LAGE3 in HCC. **(C)** The top 8 genes which were strong correlated with LAGE3 expression enriched in mTOR signaling pathway.

To predict the potential functions and pathways of LAGE3 in HCC, we chose co-expressed genes which had strong correlation with LAGE3 for GO and GSEA analysis. The results of GO biological process showed that these genes were involved in multiple processes, such as oxidative phosphorylation. GO cellular component analysis showed the cellular structures of these genes, such as mitochondrial inner membrane. GO terms revealed several molecular functions, such as catalytic activity, acting on RNA (**Figure 5A**).

In addition, the GSEA-KEGG enrichment analysis showed that these genes were closely linked to neurodegenerative diseases, such as Huntington disease and Alzheimer disease (**Figure 5B**). More importantly, besides pathways of neurodegeneration-multiple diseases, these genes were also linked to mTOR signaling pathway (**Figure 5B**). Meanwhile, the genes enriched in mTOR signaling pathway were predominantly positively correlated with LAGE3 in HCC (**Figure 5C**).

### LAGE3 Expression Was Correlated With Immune Cell Infiltration and Markers of T Cell Exhaustion in HCC

**Figure 6:**
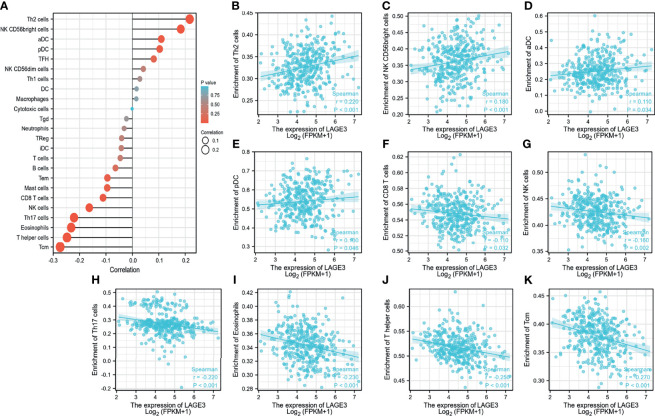
LAGE3 expression correlates with immune cell infiltration in HCC. **(A)** From TCGA database, the correlation of LAGE3 expression with 24 types of immune cells in HCC based on the Gene Set Variation Analysis (GSVA). **(B-K)** From TCGA database, the correlation of LAGE3 expression with **(B)** Th2 cells; **(C)** CD56bright NK cells; **(D)** aDC; **(E)** plasmacytoid DC; **(F)** CD8 cells; **(G)** NK cells; **(H)** Th17 cells; **(I)** eosinophils; **(J)** T helper cells, and **(K)** Tcm. Wilcoxon signed-rank test was used for statistical analysis. aDC, activated DC; Tcm, T central memory cells.

We explored the correlation between LAGE3 expression with 24 types of immune cells in HCC (23). Based on the Gene Set Variation Analysis (GSVA) (24), high expression of LAGE3 was significantly and positively correlated with T helper (Th)2 cells, CD56bright NK cells, activated dendritic cells (aDCs), and plasmacytoid DCs (**Figures 6A** **–E**). Conversely, high expression of LAGE3 was significantly negatively correlated with CD8 cells, NK cells, Th17 cells, eosinophils, T helper cells, and T central memory cells (Tcm) (**Figures 6A** **, F–K**). In addition, according to the GEPIA and TIMER databases, a positive correlation was found between the expression of LAGE3 and levels of immune checkpoints, including PD-1, CTLA-4, TIGIT, and TIM-3 (**Table 3**).

**Table 3:** Spearman correlation analysis between LAGE3 and markers of T cell exhaustion in patients with HCC by TIMER and GEPIA.

Cell type	Gene marker	TIMER		GEPIA	
		Cor	p	R	p
T cell exhaustion	PD-1 (PDCD1)	0.146	**	0.1	*
	CTLA4	0.158	**	0.11	*
	LAG3	0.133	*	0.12	*
	TIM-3 (HAVCR2)	0.123	*	0.087	0.096

*P < 0.05; **P < 0.01.

### Knockdown of LAGE3 in HCC Cell Lines

**Figure 7:**
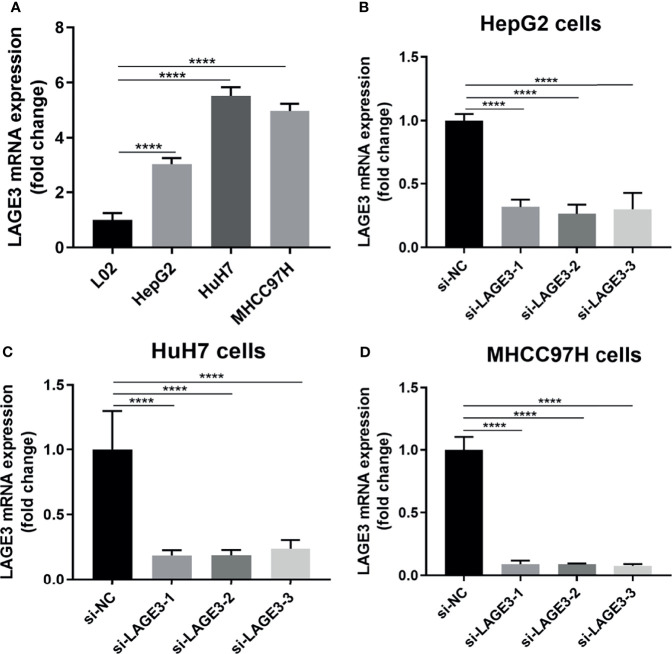
Successful knockdown of LAGE3 expression in HCC cell lines. qRT-PCR was used to detect the expression of LAGE3 mRNA. **(A)** mRNA expression level of LAGE3 in L02, HepG2, HuH-7, and MHCC97H cells. **(B-D)** mRNA expression of LAGE3 in HepG2, HuH-7, and MHCC97H cells transfected with si-LAGE3-1, si-LAGE3-2, si-LAGE3-3 or si-NC. All experiments were conducted in triplicate. Data are expressed as mean ± standard deviation (SD). ****P < 0.0001 compared with L02 or corresponding control groups.

To further verify the functions of LAGE3 on HCC cell lines, we first examined the expression of LAGE3 in three HCC cell lines (HepG2, HuH-7 and MHCC97H), and the results showed that the expression of LAGE3 was significantly higher in HCC cells than that in the normal hepatic cell line (L02) (**Figure 7A**). Next, LAGE3 expression was silenced by transfecting siRNAs in HCC cell lines and the efficiency of LAGE3 knockdown was validated by qRT-PCR in HepG2, HuH-7 and MHCC97H cell lines (**Figures 7B–D**). In particular, the si-LAGE3-2 was most effective in knocking down the expression of LAGE3. Thereby si-LAGE3-2 was selected for the subsequent studies.

### LAGE3 Levels Were Positively Associated With Proliferation and Anti-Apoptotic Abilities in HCC Cells

**Figure 8:**
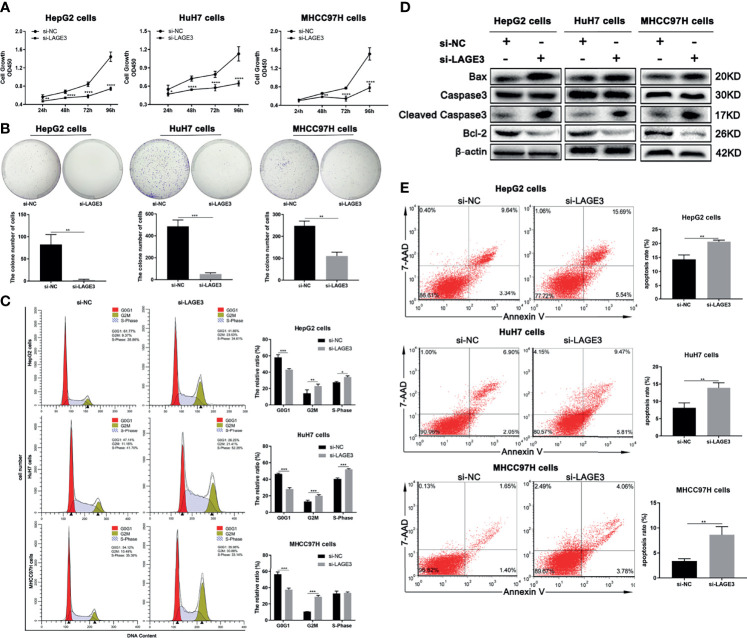
LAGE3 level is positively associated with proliferation and anti-apoptotic abilities in HCC cells. **(A)** The proliferation ability of HepG2, HuH-7, and MHCC97H cells transfected with si-LAGE3 or si-NC detected by the CCK-8 assay. **(B)** The colony formation assay detected the clone number of HepG2, HuH-7 and MHCC97H cells transfected with si-LAGE3 or si-NC. **(C)** Cell cycle analysis by flow cytometry showing the changes of G2/M and S cell cycle after LAGE3 knockdown in HepG2, HuH-7, and MHCC97H cells. **(D)** Western blots showing protein expression of caspase-3, cleaved caspase-3, and Bcl-2 in si-LAGE3 transfected HepG2, HuH-7 and MHCC97H cells respectively. **(E)** Flow cytometry was used to detect the ratio of apoptotic cells in si-LAGE3 transfected HepG2, HuH-7, and MHCC97H cells, respectively. All experiments were conducted in triplicate. Data are expressed as mean ± standard deviation (SD). *P < 0.05, **P < 0.01, ***P < 0.001 and ****P < 0.0001 compared with si-NC group.

To elucidate the possible function of LAGE3 in regulating the proliferation of HCC cells, CCK-8 assay and colony formation assays were performed. The results showed that decreased cell proliferation was found in si-LAGE3 transfected HCC cell lines, including HepG2, HuH-7 and MHCC97H cells, compared with the NC groups (**Figure 8A**). Similarly, the colony formation assay also showed that the number of clones of HepG2, HuH-7 and MHCC97H transfected with si-LAGE3 was significantly lower than that of the NC groups (**Figure 8B**). In addition, the cell cycle assay was performed to evaluate LAGE3-mediated promotion of proliferative activity by flow cytometry. All three tested HCC cell lines revealed a G2/M or S cell cycle arrest after LAGE3 knockdown (**Figure 8C**). These data suggested that LAGE3 was important for the proliferation of HCC cells.

To verify the effects of LAGE3 on HCC cell apoptosis, we evaluated the expression of key proteins involved in cell apoptosis. The increased expression of cleaved caspase-3 and Bax, as well as decreased expression of Bcl-2 were detected in si-LAGE3 transfected HepG2, HuH-7 and MHCC97H cells, compared with NC groups (**Figure 8D**). Furthermore, flow cytometry also showed that the number of apoptotic cells increased markedly in si-LAGE3-transfected HepG2, HuH-7 and MHCC97H cells compared with the NC groups (**Figure 8E**). These results indicated that LAGE3 played an anti-apoptotic role in HCC.

### Down-Regulation of LAGE3 Suppressed Migration and Invasion of HCC Cell Lines

**Figure 9:**
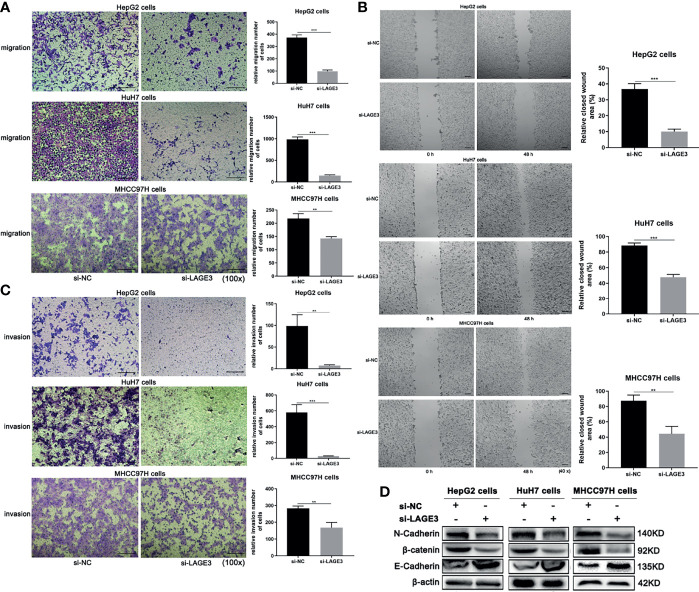
Down-regulation of LAGE3 suppresses migration and invasion of HCC cell lines. **(A, B)** Transwell assays **(A)** and wound-healing assays **(B)** showing the migration of HepG2, HuH-7, and MHCC97H cells transfected with si-LAGE3 or si-NC, respectively. **(C)** The invasion abilities of HepG2, HuH-7, and MHCC97H cells transfected with si-LAGE3 or si-NC. **(D)** Changes in protein expression of E-cadherin, N-cadherin, and β-catenin in HepG2, HuH-7, and MHCC97H cells transfected with si-LAGE3 or si-NC. All experiments were conducted in triplicate. Data are expressed as mean ± standard deviation (SD). **P < 0.01 and ***P < 0.001 compared with si-NC group.

To explore the role of LAGE3 in cell migration and invasion, the Transwell and wound-healing assays were performed. Migration capacity was significantly lower in si-LAGE3 transfected HepG2, HuH-7 and MHCC97H cells than in the NC groups (**Figures 9A, B** **)**. Moreover, compared with corresponding controls, the invasions of si-LAGE3-transfected HepG2, HuH-7 and MHCC97H cells were attenuated (**Figure 9C**), indicating that LAGE3 inhibited the invasion of HCC cells. In addition, compared to the si-NC groups, the up-regulation of E-cadherin and down-regulation of N-cadherin, β-catenin were observed in si-LAGE3 transfected groups (**Figure 9D**), further supporting the regulatory roles of LAGE3 in mediating HCC cell migration and invasion. Taken together, these results confirmed that LAGE3 promoted cell migration and invasion capacities of HCC.

### LAGE3 Knockdown Attenuated the Tumorigenicity of HCC Cell Lines *In Vivo*

**Figure 10:**
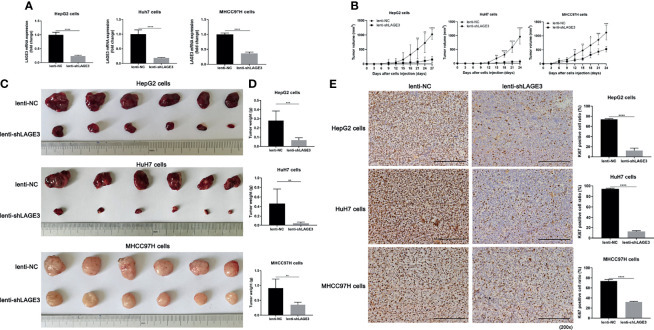
LAGE3 knockdown attenuates the tumorigenicity of HCC cell lines *in vivo*. **(A)** mRNA expression of LAGE3 in HepG2, HuH7 and MHCC97H cells transfected with lenti-NC or lenti-shLAGE3. **(B)** Growth curves of xenograft tumors in nude mice of lenti-NC groups and lenti-shLAGE3 groups. The tumor volume was calculated using the formula: V = L×W^2^/2 (L = tumor length, W = tumor width). **(C)** Images of HepG2, HuH7 and MHCC97H xenograft tumors in nude mice of lenti-NC groups and lenti-shLAGE3 groups. **(D)** Comparison of the tumor weight between lenti-NC groups and lenti-shLAGE3 groups. **(E)** Immunohistochemical staining of Ki67 in xenograft tumor tissues from lenti-NC groups and lenti-shLAGE3 groups. All of the data are expressed as mean ± SD. n = 6 for BALB/c-nu nude mice following each treatment per experimental xenograft. *P < 0.05, **P < 0.01, ***P < 0.001 and ****P < 0.0001 compared with lenti-NC group.

To further explore the role of LAGE3 *in vivo*, the HepG2, Huh7 and MHCC97H cells were used to establish xenograft tumor models by injecting cells transfected with lenti-NC or lenti-shLAGE3, respectively. qRT-PCR results successfully validated the efficiency of lenti-shLAGE3 infection with HepG2, Huh7 and MHCC97H cells (**Figure 10A**). Accordingly, the results from the subcutaneous tumor-bearing mice models showed the lentivirus-mediated silencing of LAGE3 by shRNA reduced tumor volumes and weights, compared to the lenti-NC mice (**Figures 10B** **–D**). Moreover, as presented in **Figure 10E**, IHC revealed that the expression of Ki67, a protein strongly associated with tumor cell proliferation and growth, was markedly reduced in tumor tissues of HepG2, Huh7 and MHCC97H with LAGE3 knockdown, compared with corresponding lenti-NC tissues. Thus, these findings further indicated that LAGE3 accelerated the tumorigenicity of HCC cell lines *in vivo*.

### LAGE3 Knockdown Could Enhance the Sensitivity of Tumor Cell Death Induced by Cytotoxic T Cells

**Figure 11:**
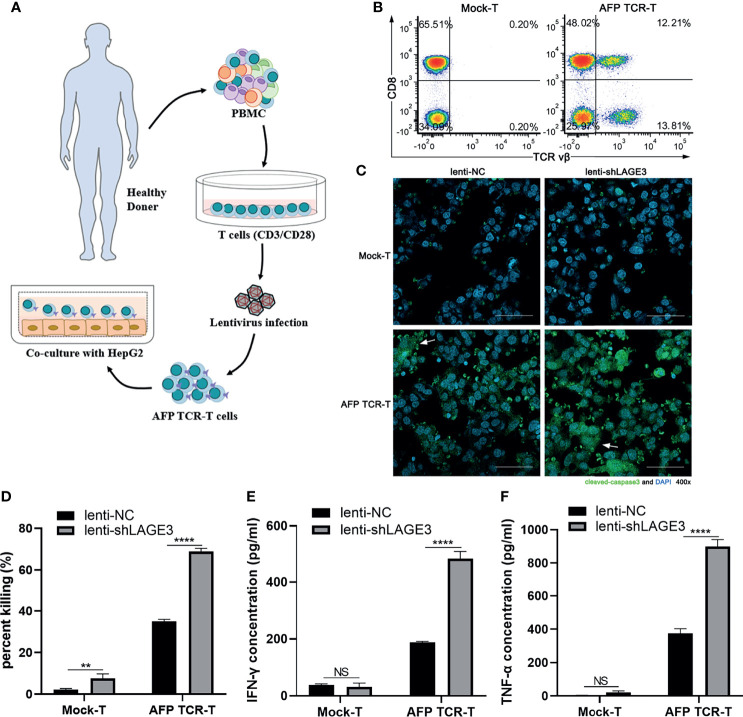
LAGE3 knockdown could enhance the sensitivity of cell death induced by cytotoxic T cells. **(A)** The flowchart of engineering AFP TCR-T cells. **(B)** Flow cytometry was used to detect the ratio of AFP specific cytotoxic T cells (TCR vβ^+^CD8^+^) in AFP TCR-T cells. **(C)** The cleaved caspase-3 expression of HepG2 transfected with lenti-NC or lenti-shLAGE3 detected using immunofluorescence. **(D)** The killing ratio assessed by detecting LDH in co-culture supernatant. **(E, F)** The levels of IFN-γ and TNF-α in co-culture supernatant. **P < 0.01, ****P < 0.0001 compared with corresponding control groups. NS, no significance.

To verify the effects of LAGE3 expression on tumor cell death induced by cytotoxic T cells, AFP TCR-T cells were constructed using gene engineering technology. The flowchart of engineering AFP TCR-T cells was shown (**Figure 11A** **)**. The ratio of TCR vβ^+^CD8^+^ cells indicated that the ratio of AFP specific cytotoxic T cells in AFP TCR-T cells was 12.1% (**Figure 11B**). Furthermore, AFP TCR-T cells were cocultured with HepG2 cells infected with lenti-NC or lenti-shLAGE3 at 2.5:1. After 3 h, the immunofluorescence experiment found that the expression of cleaved caspase-3 was significantly increased in HepG2 cells infected with lenti-shLAGE3, compared with those infected with lenti-NC from AFP TCR-T groups (**Figure 11C**). In addition, after cocultured with AFP TCR-T cells for 12 h, the cell killing percent as well as secretion levels of IFN-γ and TNF-α were also higher in the group of HepG2 cells with LAGE3 knockdown, compared with those of NC group (**Figures 11D** **–F**).

## Discussion

Despite the great progress in the development of multiple therapies over the years, the prognosis of patients with advanced HCC remains poor. At present, early diagnosis is also a key strategy to improve the prognosis of HCC, especially in the patients who are asymptomatic and have sufficient liver function (25). Nonetheless, to date, diagnostic, prognostic or therapeutic biomarkers for HCC are still inadequate. Therefore, it is urgent to identify effective biomarkers of HCC to guide more optimized diagnostic and therapeutic strategies.

LAGE3 was described as an important component of the complex responsible for forming the N^6^-threonylcarbamoyladenosine (t^6^A) moiety in position 37 of tRNAs (26). Studies have shown that LAGE3, an RNA modification-related protein, is most frequently upregulated in multiple cancer types (10). In addition, it was found that the down regulation of LAGE3 significantly affected the biological function of some cancer cells, including PTC and BC cell lines (14, 16). However, the functions and regulatory mechanisms of LAGE3 are not fully clear in HCC. Thus, our study provides new evidence for the clinical value, potential biological roles and regulatory mechanisms of LAGE3 in HCC.

Studies have found that LAGE3 was overexpressed in multiple tumor tissues compared with normal tissues (11–16). Similarly, our study also found that the levels of LAGE3 gene and protein expression were significantly higher in HCC tumor tissues than in normal tissues through public online tools. Additionally, the high LAGE3 expression was significantly associated with poor prognosis in HCC. These data indicated that LAGE3 may be suitable as a potential prognostic marker for HCC. The ROC curve analysis also verified the diagnostic value of LAGE3 in HCC. However, these results need to be further analyzed and validated by a larger clinical cohort.

Immune infiltration is a prognostic factor in human tumors (27). The relative proportion and types of tumor-infiltrating immune cells in the tumor microenvironment (TME) may be associated with inflammation, cancer immune evasion, and clinical outcomes of patients (28). Previous studies have found that LAGE3 could be an immune-related biomarker associated with a wide variety of cancers (11–16). The expression level of LAGE3 was correlated with the infiltration levels of different immune cells (11–16). Interestingly, in our study, based on the GSVA (24) and multiple online databases, our findings showed that high expression of LAGE3 was markedly negative correlated with the infiltration level of CD8^+^ T cells. Previous studies have shown that higher infiltration of CD8^+^T cells is associated with a favorable prognosis of multiple cancers (29, 30). Tumors with higher infiltration of CD8^+^ T cells are more likely to benefit from immunotherapy than those with lower infiltration of CD8^+^ T cells (31). Therefore, HCC patients with lower LAGE3 expression might be more susceptible to immunotherapy, however this hypothesis requires further experimental and clinical validation.

A deeper analysis of the complexity within the TME may help to identify potential biomarkers that would facilitate the identification of patients responding to recent immune checkpoint therapy strategies (32). According to **Table 3**, our study showed a positive correlation between LAGE3 expression and PD-1, CTLA-4, LAG3, and TIM-3, which are four of the most typical exhaustion markers of T cells. Currently, immune checkpoint blockade targeting the PD-L1/PD-1 axis and CTLA-4 has revealed promising clinical effects (33). In addition, targeting TIM-3 treatment could promote antitumor immunity mediated by T cell and suppress established tumors (33, 34). Therefore, our results indicated the potential mechanisms involved in LAGE3 regulation of T cell functions in HCC. Furthermore, validation of our coculture experiments using AFP specific TCR-T cells showed that knockdown of LAGE3 in HepG2 cells could increase the sensibility of tumor cell death induced by cytotoxic T cells and the ability of cytokine secretion of cytotoxic T cells. These results suggested that LAGE3 could inhibit the HCC cell death mediated by cytotoxic T cells.

The biological functions of tumor cells play an important role in the occurrence and development of tumors (35). Studies have found that down-regulation of LAGE3 may significantly reduce the proliferation, migration and invasion capacity of PTC and BC cells (14, 16). Moreover, previous studies have reported that long non-coding RNA NEAT1 influenced the proliferation and migration of HCC cells by targeting LAGE3 (36). Furthermore, one study found that LAGE3 promoted cell proliferation, migration, and invasion and inhibited cell apoptosis of HCC by facilitating the JNK and ERK signaling pathway through Hep-3B and SK-HEP1 cell lines (17). Similarly, in our study, we verified that LAGE3 knockdown significantly inhibited the proliferation, migration, and invasion of HCC using *in vivo* and *in vitro* experiments, which further verified the oncogenic potential of LAGE3 in HCC. Thus, HCC patients may achieve potential benefits from LAGE3 targeted therapy.

Both the biological functions of tumor cells and the tumor immunosuppressive microenvironment are critical for HCC treatment. Thus, the combinations of molecularly targeted therapies and immunotherapies are emerging to boost the immune responses against HCC (37). Currently, in addition to the advancements in biomarker-driven treatments, multiple immunotherapies, such as checkpoint inhibitors, inhibitory cytokine blockade, oncolytic viruses, adoptive cellular therapies and vaccines are being extensively studied or in clinical trials (33). In addition to affecting the biological function of HCC cell lines, the LAGE3 expression may also be involved in regulating the TME of HCC. Therefore, LAGE3 may be a promising therapeutic target for combination with various available immunotherapies in the near future.

To further study the potential downstream cascades of LAGE3 in HCC, the PPI network analysis was done. And it showed that LAGE3 could interact with TP53RK and ILF2 in HCC. Studies have shown that TP53RK is becoming a potential novel target in multiple myeloma. Inhibition of TP53RK could trigger multiple myeloma cell apoptosis *via* both the p53-Myc axis-dependent and independent pathways (38). In addition, ILF2 directly binds to cyclic adenosine monophosphate response element-binding protein (CREB), and this binding is essential for the malignant phenotypes of liver cancer cells (39). Thus, the mechanism of promoting HCC progression by LAGE3 may be involved in the TP53RK or ILF2-mediated pathways.

In addition, the mTOR signaling pathway could be enriched in the co-expressed genes which had strong correlation with LAGE3 using the GSEA-KEGG enrichment analysis. The roles of Akt/mTOR pathway has been extensively studied in HCC. The mTOR signaling activation could promote HCC tumorigenesis and lung metastasis (40). And the mTOR inhibitors everolimus and sirolimus could suppress cell proliferation and tumor growth in animal models of HCC (41). Therefore, we speculated that LAGE3 may play roles *via* mTOR signaling pathway. Furthermore, our study found that the changes of LAGE3 levels could regulate the expression of N-cadherin, β-catenin, and E-cadherin in HCC cell lines. β-catenin, a transcription factors, is a direct repressor of E-cadherin expression (42). E-cadherin and N-cadherin are two classical cadherins related to epithelial-to-mesenchymal transition (EMT), a crucial regulatory mechanism of tumor cell migration and invasion in multiple cancers, including HCC (43–45). The switch from E-cadherin to N-cadherin is a vital sign of EMT induction. Interestingly, the mTOR signaling pathway is one of the most important signaling pathways during EMT process in multiple cancers, including HCC (46, 47). Hence, the mTOR pathway may be one of the potential downstream cascades by which LAGE3 regulates the EMT process in HCC cells. However, more experiments are needed to verify this conclusion.

## Conclusion

In summary, our results indicated that LAGE3 was upregulated in HCC, and higher LAGE3 expression was correlated with shorter OS, worse prognosis, and was associated with infiltration of numerous immune cells in HCC patients. Besides, LAGE3 was also involved in regulating numerous biological functions of HCC cell lines, including proliferation, apoptosis, migration, invasion, the sensitivity of tumor cell death. These findings suggested that LAGE3 played a significant role in the progression of HCC. Thus, LAGE3 may be considered as a potential biomarker and therapeutic target.

## Data Availability Statement

The original contributions presented in the study are included in the article. Further inquiries can be directed to the corresponding authors.

## Ethics Statement

The animal study was reviewed and approved by The Animal Ethics Committee of Nanfang Hospital of Southern Medical University (China).

## Author Contributions

WZ, XZ, and JH were responsible for the conception and design of the study. QC, XL, JX, and WX designed and performed experiments. QC, XL, WX, JX, ZZ, NM, XH, and HL participated in data acquisition and analysis. QC wrote the manuscript. WZ and XZ were involved in revising the manuscript. All authors provided final approval of the version to be published.

## Funding

This work was supported by the National Natural Science Foundation of China (82073360, 81802449) and China Postdoctoral Science Foundation (2020M672739, 2020T130281).

## Conflict of Interest

The authors declare that the research was conducted in the absence of any commercial or financial relationships that could be construed as a potential conflict of interest.

## Publisher’s Note

All claims expressed in this article are solely those of the authors and do not necessarily represent those of their affiliated organizations, or those of the publisher, the editors and the reviewers. Any product that may be evaluated in this article, or claim that may be made by its manufacturer, is not guaranteed or endorsed by the publisher.
